# The effects of vitamin D supplementation on serum lipid profiles in people with type 2 diabetes: a systematic review and meta-analysis of randomized controlled trials

**DOI:** 10.3389/fnut.2024.1419747

**Published:** 2024-06-05

**Authors:** Qingyang Lu, Qingyue Liang, Yue Xi

**Affiliations:** ^1^School of Public Health, The University of Hong Kong, Pokfulam, Hong Kong SAR, China; ^2^Discipline of Nutrition and Dietetics, Faculty of Medicine and Health, The University of Sydney, Sydney, NSW, Australia; ^3^Centre for International Collaboration, Office of Hong Kong, Macao and Taiwan Affairs, Sir Run Run Shaw Hospital, Zhejiang University School of Medicine, Hangzhou, China

**Keywords:** type 2 diabetes, vitamin D supplementation, lipid profiles, meta-analysis, cardiovascular diseases

## Abstract

**Introduction:**

People with type 2 diabetes (T2D) are highly susceptible to the development of cardiovascular diseases. Previous studies have suggested that the application of vitamin D may offer potential benefits in improving lipid profiles, but these effects remain controversial.

**Methods:**

This systematic review and meta-analysis focused on the effects of vitamin D supplementation on serum lipid profiles in people with T2D. Randomized controlled trials (RCTs) assessing the effects of vitamin D supplementation on lipid profiles and published before September 19th, 2023, were identified in PubMed, Embase, and Cochrane Library. This review protocol was registered in the PROSPERO (CRD42023461136). The random-effects model was employed to estimate unstandardized mean differences (MD) and 95% confidence intervals (CIs). The quality of studies was assessed by the Cochrane Risk of Bias tool 2.

**Results:**

Overall, 20 RCTs involving 1711 participants were included. Results indicated that vitamin D supplementation significantly improves serum high-density lipoprotein (HDL) (MD: 1.63 mg/dL, 95% CI: 0.19 to 3.08, P = 0.03), and triglyceride (TG) levels (MD: -8.56 mg/dL, 95% CI: -15.23 to -1.89, P = 0.01). However, vitamin D supplementation failed to improve low-density lipoprotein (LDL) levels and total cholesterol (TC) levels. Subgroup analyses and meta-regressions suggested that higher doses of vitamin D supplementation and shorter duration of intervention were more likely to have favorable effects on lipid profiles. Moreover, participants with lower baseline BMI and higher serum 25-hydroxy vitamin D levels exhibited greater improvements in lipid profiles following vitamin D supplementation.

**Conclusions:**

This meta-analysis highlighted the effects of vitamin D supplementation on improving serum HDL and TG levels while not exhibiting significant improvements in LDL and TC levels. Further long-term and high-quality studies are still needed to draw more precise conclusions.

**Systematic review registration:**

https://www.crd.york.ac.uk/prospero/display_record.php?RecordID=461136.

## 1 Introduction

Type 2 Diabetes (T2D) has emerged as a significant public health concern globally, with its prevalence expected to rise from 536.6 million in 2021 to 780 million by 2045 (1). People with T2D are at an elevated risk of developing cardiovascular diseases (CVD). The incidence of CVD among people with T2D is 2 to 3 times higher compared to non-diabetic persons (2). Dyslipidemia is frequently observed in people with T2D, which is also a widely recognized risk factor for CVD. It is characterized by raised levels of serum total cholesterol (TC) and low-density lipoprotein (LDL) and decreased high-density lipoprotein (HDL) levels (3).

Vitamin D is a fat-soluble vitamin that naturally occurs in a limited number of foods and can be acquired through dietary supplementation (4). It is essential for maintaining bone health and has additional benefits for extra-skeletal effects such as regulation of inflammation (4, 5). Auto-immune activation and low-grade inflammation play significant roles in the onset and progression of T2D, as increased inflammatory cytokine activity would cause beta-cell death in the pancreas and raise insulin resistance in target cells (6). Similarly, vitamin D may lower the risks of CVD via the mechanism of down-regulating inflammation and increase insulin sensitivity (7), indicating its potential to prevent both T2D and CVD.

Vitamin D status is determined by serum 25-hydroxy vitamin D (25OHD) concentration. The Endocrine Society defines vitamin D status based on serum levels of 25OHD, with deficiency indicated by less than 50 nmol/L (8, 9). People with T2D may have lower serum 25OHD levels and thus be more susceptible to vitamin D deficiency (10). Moreover, deficient vitamin D levels may also be associated with unfavorable serum lipid profiles, particularly TC, LDL and HDL levels (11).

The effects of vitamin D on lipid profiles among people with T2D have been investigated in these two decades. A previous meta-analysis conducted by Jafari et al. (6) in 2016 demonstrated a significant reduction in serum LDL and TC levels with the administration of vitamin D, while no significant effect was observed on triglyceride (TG) levels. The effects on serum HDL levels were statistically significant but the absolute difference was negligible (6). Through their subgroup analyses, only doses of vitamin D less than 2000 IU significantly decrease TG and TC levels, while only interventions lasting less than 12 weeks significantly reduce LDL and HDL levels (6). However, recent randomized controlled trials (RCTs) have produced inconsistent results compared to the findings of the previous meta-analysis. Hu et al. (12) revealed no significant change in LDL, HDL and triglycerides (TG) levels by supplementation with 800 IU vitamin D per day for 30 months. Nevertheless, El Hajj et al. (13) demonstrated that administration of 4,000 IU/day of vitamin D, for 6 months, significantly reduced TG level and increased HDL level, but insignificantly changed levels of LDL and TC. Therefore, further consensus regarding the impact of vitamin D supplementation on lipid profiles remains to be established.

In addition, the meta-analysis conducted by Jafari et al. (6) also pointed out that vitamin D fortification may yield more favorable effects on lipid profiles compared to supplementation. Given that vitamin D fortification often employs lower doses and shorter intervention periods (14, 15), the effects of vitamin D (fortification and supplementation) on lipid profiles may differ compared to solely administering vitamin D supplementation. However, to our knowledge, there is currently no existing meta-analysis purely focused on investigating the impact of vitamin D supplementation on lipid profiles among individuals with T2D. As such, we performed a systematic review and meta-analysis specifically focused on vitamin D supplementation to examine its effects on lipid profiles (TC, TG, LDL, and HDL) in people with type 2 diabetes. Our secondary outcomes involve examining the influence of variables such as dose, duration, baseline 25 OHD levels, BMI, type of vitamin D supplementation, and publication year on the effects of vitamin D supplementation on lipid profiles.

## 2 Materials and methods

The protocol has been registered in the international Prospective Register of Systematic Reviews (PROSPERO, registration number: CRD42023461136). This review was performed based on Preferred Reporting Items for Systematic Reviews and Meta-Analyses (PRISMA) guidelines (16).

### 2.1 Literature search strategies

Three databases including PubMed, Embase, and Cochrane Library were searched. RCTs were selected from database inception up to September 19th, 2023. The search terms including Medical subject headings (MeSH) and non-MeSH search terms were presented as follows: ((“Vitamin D” OR “ergocalciferols” OR “Vitamin D2” OR “ergocalciferol” OR “25-hydroxyvitamin D2” OR “Dihydrotachysterol” OR “calcifediol” OR “cholecalciferol” OR “Hydroxycholecalciferols” OR “Calcitriol” OR “dihydroxycholecalciferol” OR “Calciferol”) AND (“Type 2 Diabetes Mellitus” OR “Type 2 Diabetes” OR “Noninsulin Dependent Diabetes Mellitus” OR “Diabetes, Type 2” OR “Diabetes Mellitus” OR “type 2 diabetes” OR “type 2 diabetes mellitus”) AND (“Intervention” OR “controlled trial” OR “randomized” OR “randomised” OR “placebo” OR “trial” OR “Trial” OR “trials” OR “randomized controlled trial” OR “randomised controlled trial” OR “RCT” OR “blinded” OR “double blind” OR “double blinded” OR “clinical trial” OR “Cross-Over” OR “parallel” OR “randomly”)). Moreover, we manually search references to avoid missing additional eligible studies.

### 2.2 Study selection

Two researchers (Qingyang Lu and Yue Xi) independently ascertained eligible studies by reading titles, abstracts, and full text if necessary. The inclusion studies were those written in English only. We only included parallel RCTs that provided sufficient information to examine the effects of vitamin D supplementation on lipid profiles LDL, HDL, TC, and TG in people with T2D. All types of vitamin D such as vitamin D3 (cholecalciferol), vitamin D2 (ergocalciferol), calcitriol (1, 25-hydroxyvitaminD3), and unspecified types of vitamin D treated in intervention were included. We excluded studies if: (i) studies were duplicated, conference papers, letters, reviews, animal studies, observational studies, RCTs with inappropriate control or intervention groups, or open-label RCTs; (ii) participants of studies were pre-diabetes, type 1 diabetes mellitus, gestational diabetes mellitus, or T2D with nephropathy; (iii) studies lacked adequate data in terms of lipid profiles and baseline information; (iv) studies used vitamin D fortification. Any discrepancies in the study selection process were discussed with a third researcher (Qingyue Liang).

### 2.3 Data extraction and quality assessment

We extracted data from each study including: (i) basic characteristics of studies regarding first author’s last name, publication year, study location, intervention duration, doses of vitamin D supplementation and sample size in each arm. Dose were uniformly calculated as daily dose (international unit, IU) and the longest duration of intervention was collected; (ii) basic characteristics of participants including mean age, baseline mean serum 25OHD (nmol/L), baseline body mass index (BMI) in each arm; (iii) mean and Standard Deviation (SD) of lipid profiles (at baseline and end of intervention, changes from baseline to end of intervention in each group, or changes from baseline to end of intervention between two groups). The process of data extraction was conducted by two researchers independently (Yue Xi and Qingyang Lu). We followed Cochrane guidelines and used the Cochrane Risk of Bias tool-2 (RoB 2) to assess the quality of each included RCT (17, 18). The ROB 2 tool comprises five domains and assigns judgments of “Low,” “Some concerns,” and “High” within each domain (18).

### 2.4 Data synthesis

Data of lipid profiles reported in millimoles per liter were manually converted into milligrams per deciliter by multiplying with 38.67 for LDL, HDL, and TC, and by 88.57 for TG (19). For data given adjusted coefficients and 95% confidence intervals (CI), CI was converted into SDs by using the formula $\sqrt{n}\times \frac{\left(UL-LL\right)}{3.92}$ if the sample size is over 50. For data presented as median and the first and the third quartiles, the mean was calculated based on the method proposed by Luo et al. (20) and SD was estimated based on the findings of Wan et al. (21). If the change of lipid profiles were not given directly in the original studies, the mean of changes was calculated by subtracting the baseline from end-of intervention in each group. Related SD was imputed by using $\sqrt{SD_{b}^{2}+SD_{f}^{2}-\left(2\times 0.5\times SD_{b}\times SD_{f}\right)}$, where SD_b_ was baseline SD and SD_f_ was end-of-intervention SD and the correlation coefficient was assumed as 0.5 (22).

### 2.5 Statistical analysis

The unstandardized mean difference (MD) and 95% confidence interval (CI) between the control and intervention groups were estimated using random-effects models in this meta-analysis (23). Heterogeneity between studies was estimated by Cochran’s Q test, Tau-squared (tau^2^) and I-squared (I^2^) value. The values of I^2^ below or equal to 25%, between 26 and 50%, and above 50% are denoted as low, moderate, and high heterogeneity, respectively (6). To identify resources of heterogeneity and mean differences among various factors, subgroup analyses were conducted based on baseline 25 OHD level (≥ 50 nmol/L and < 50 nmol/L), baseline BMI (≥ 30 kg/m^2^ and < 30 kg/m^2^), vitamin D doses (≥ 5,000 IU/day and < 5,000 IU/day), intervention duration (≥ 24 weeks and < 24 weeks), publication year (2016 and before, 2017 and beyond) and type of vitamin D supplementation. Moreover, meta-regression analyses were applied if variables were continuous, such as doses, duration, baseline 25 OHD and BMI. Permutation tests were additionally conducted to ensure the robustness of meta-regressions. Permutation resampling is applied to assess the absence of an effect in scenarios where there is uncertainty regarding the distribution of the test statistic or when the data are not randomly sampled from a defined population (24). We also performed the leave-one-out analysis as a sensitivity analysis of heterogeneity. In addition, publication bias was detected by using the contour-enhanced funnel plot, Egger’s test, and Begg’s test. The plots of quality assessments were conducted by using the R package “robvis” (25). The statistical analysis was conducted by using R software, Version 4.3.0. All statistical tests were performed as two-sided, and the significance level was set at *p* < 0.05.

## 3 Results

### 3.1 Study selection

**Figure 1:**
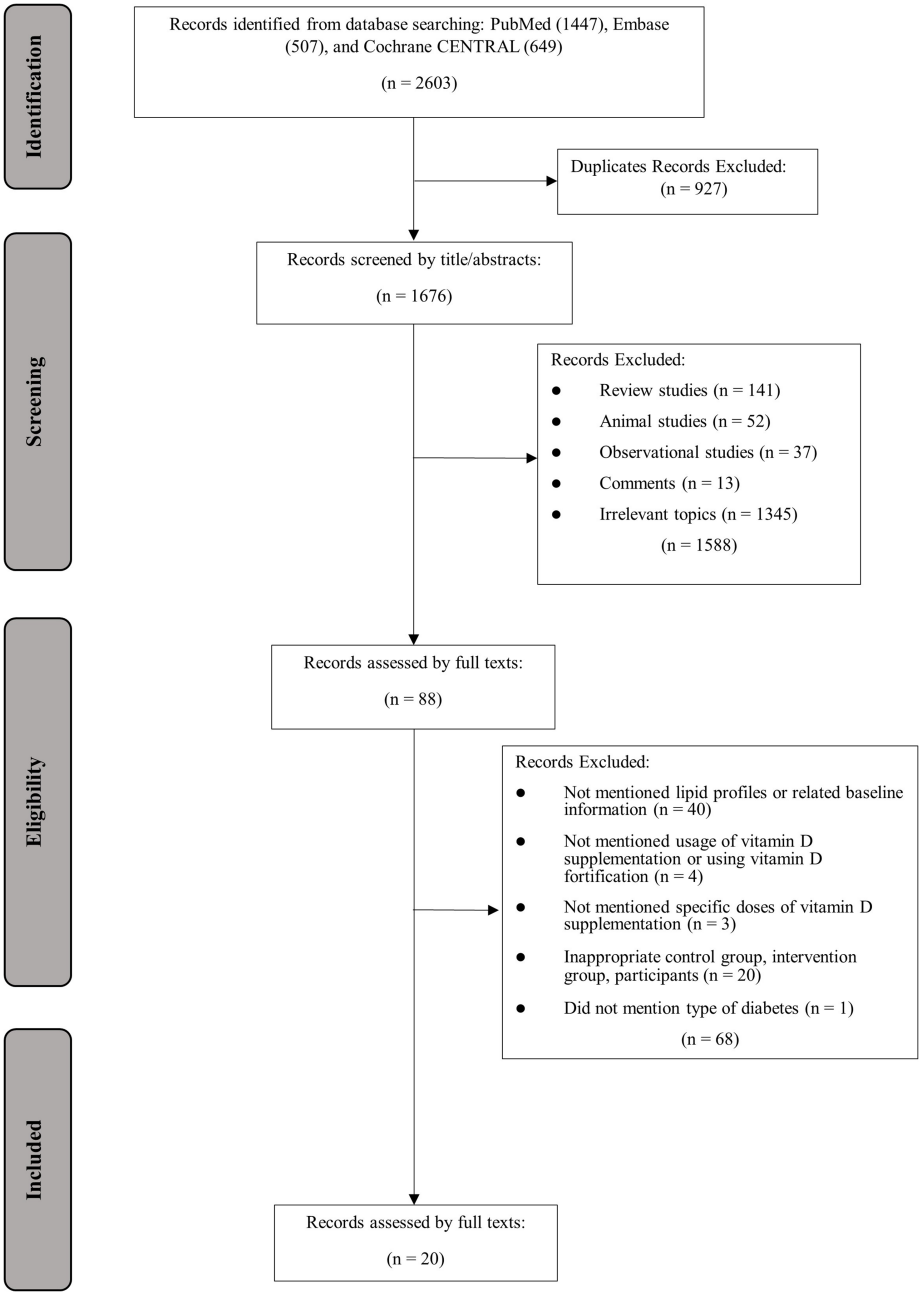
The PRISMA flowchart of study search, screening, selection, and inclusion.

The process of study selection and identification is presented in Figure 1. In total, 2,603 articles were found in database searching, out of which 927 were identified as duplicates. The remaining 1,676 articles were screened by title and abstract, with 1,588 articles excluded due to irrelevance to the topic or other reasons. Eighty-eight articles were assessed for eligibility, and an additional 68 studies were excluded due to the following reasons: lacked information on lipid profiles, usage or doses of vitamin D supplementation, baseline 25OHD or BMI, or administration of vitamin D fortification (*n* = 47); inappropriate control or intervention group (*n* = 20); not mention the specific type of diabetes (*n* = 1). Finally, 20 articles with 24 effect sizes were included (12, 26–44).

### 3.2 Study characteristics

**Figure 2:**
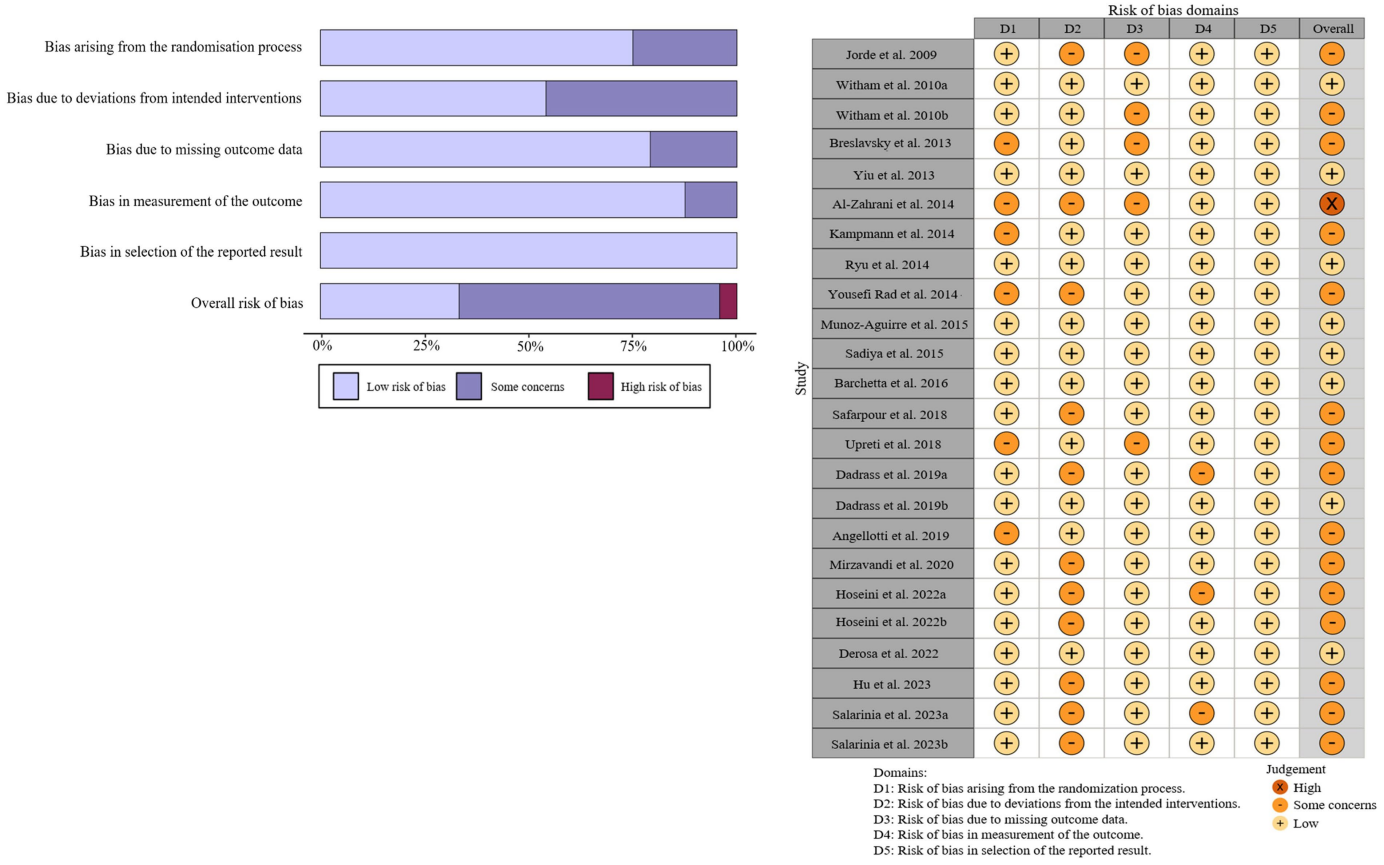
Summary **(A)** and graph **(B)** of the risk of bias in the selected studies by the Cochrane Risk of Bias tool-2 (ROB2).

Detailed characteristics of included studies are presented in Table 1. Overall, most of the studies (n = 13) were conducted in Asia (12, 26, 29, 30, 32, 33, 35, 37–39, 41, 42, 44). Thirteen studies clarified using vitamin D3 as their supplementation (12, 26–32, 34–36, 40, 43), while the remaining 7 studies did not specify the type of vitamin D (33, 37–39, 41, 42, 44). A total of 1711 participants were admitted into these 20 studies, with 861 participants in the intervention group and 851 participants in the control group. At baseline, the mean age of all participants was 54.9 years (range: 42.3 to 66.3 years), the average BMI was 29.6 kg/m2 (range: 24.7 to 37.8 kg/m2) and the average level of 25 OHD was 46.6 nmol/L (range: 23.6 to 86.8 nmol/L). The duration of the intervention ranged from 8 to 120 weeks, with an average of 22.2 weeks, while the dosage of vitamin D supplementation varied from 800 to 7142.9 IU/day, with a mean of 4341.27 IU/day. The quality assessments indicated that most of the included studies had low quality or were discovered with some concerns, except of the study conducted by Al-Zahrani et al. (26), which was deemed to have high risks. The detailed results of the quality assessment are presented in Figure 2.

**Table 1:** Baseline characteristics of included RCTs in the meta-analysis.

Study	Country	Type of vitamin D	Sample size		Doses (IU/day)	Duration (week)	Baseline information of participants			Intervention	
			IG	CG			Age (years)	BMI (kg/m^2^)	25 OHD (nmol/L)	IG	CG
Jorde et al. (27)	Norway	Vitamin D3	16	16	5714.29	24	56.25	32.05	59.25	Vitamin D	Placebo
Witham et al. (28)	United Kingdom	Vitamin D3	19	21	833.33	16	66.04	32.26	43.10	Vitamin D	Placebo
Witham et al. (28)	United Kingdom	Vitamin D3	18	21	1666.67	16	65.13	31.64	46.38	Vitamin D	Placebo
Breslavsky et al. (29)	Israel	Vitamin D3	24	23	1000.00	48	66.31	28.73	29.69	Vitamin D	Placebo
Yiu et al. (30)	China	Vitamin D3	50	50	5000.00	12	65.35	25.45	53.75	Vitamin D	Placebo
Al-Zahrani et al. (26)	Saudi Arabia	Vitamin D3	91	92	4821.43	12	54.69	31.65	23.64	Vitamin D	No treatment
Kampmann et al. (31)	Denmark	Vitamin D3	7	8	6533.33	12	59.15	33.75	33.03	Vitamin D	Placebo
Ryu et al. (32)	South Korea	Vitamin D3	32	30	2000.00	24	55.56	24.84	28.81	Vitamin D + calcium	Placebo + calcium
Yousefi Rad et al. (33)	Iran	Not specified	28	30	4000.00	8	49.96	28.36	37.70	Vitamin D	Placebo
Muñoz-Aguirre et al. (34)	Mexico	Vitamin D3	52	52	4000.00	24	56.75	30.65	54.55	Vitamin D	Placebo
Sadiya et al. (35)	Ajman	Vitamin D3	43	39	4500.00	24	48.52	37.76	29.45	Vitamin D	Placebo
Barchetta et al. (36)	Italy	Vitamin D3	26	29	2000.00	24	58.67	30.09	43.93	Vitamin D	Placebo
Safarpour et al. (37)	Iran	Not specified	43	42	7142.86	8	50.20	30.89	43.50	Vitamin D	Placebo
Upreti et al. (38)	India	Not specified	30	30	3750.00	24	49.10	25.07	25.99	Vitamin D	Placebo
Dadrass et al. (39)	Iran	Not specified	12	12	3571.43	12	54.33	28.47	35.88	Vitamin D + resistance training	Resistance training
Dadrass et al. (39)	Iran	Not specified	12	12	3571.43	12	53.50	27.75	37.88	Vitamin D	Placebo
Angellotti et al. (40)	United States	Vitamin D3	59	61	4000.00	48	60.20	30.95	66.50	Vitamin D	Placebo
Mirzavandi et al. (41)	Iran	Not specified	25	25	7142.86	8	45.70	29.91	40.00	Vitamin D	No treatment
Hoseini et al. (42)	Iran	Not specified	10	10	7142.86	8	47.73	29.48	54.59	Vitamin D + aerobic training	Aerobic training
Hoseini et al. (42)	Iran	Not specified	10	10	7142.86	8	48.69	28.74	58.09	Vitamin D	Placebo
Derosa et al. (43)	Italy	Vitamin D3	119	113	3571.43	24	54.47	27.52	45.99	Vitamin D	Placebo
Hu et al. (12)	China	Vitamin D3	115	105	800.00	120	66.08	24.71	57.05	Vitamin D	No treatment
Salarinia et al. (44)	Iran	Not specified	10	10	7142.86	8	42.30	29.90	86.75	Vitamin D + water exercise	Water exercise
Salarinia et al. (44)	Iran	Not specified	10	10	7142.86	8	43.30	29.94	84.38	Vitamin D	No treatment

25 OHD: 25-hydroxyvitamin D; IG: intervention group; CG: control group.

### 3.3 Effect of vitamin D supplementation on serum LDL level

**Figure 3:**
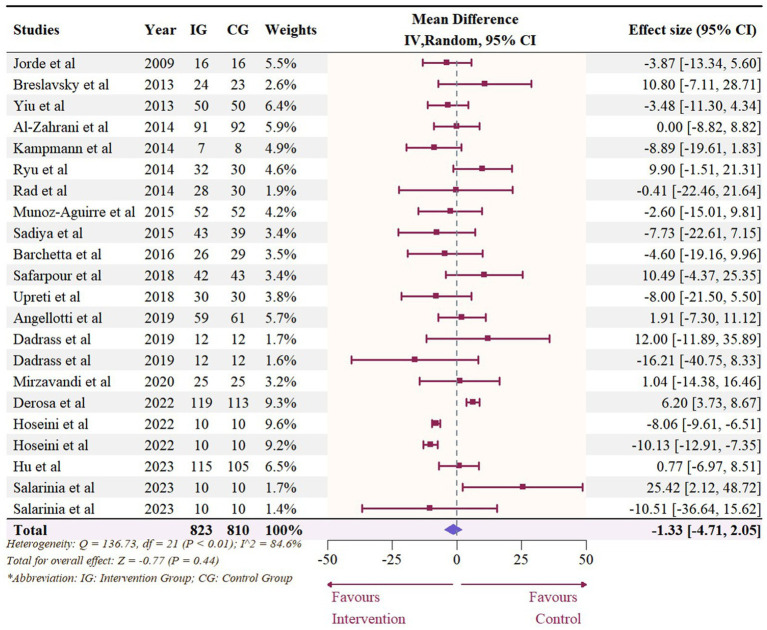
Forest plot of the effect of vitamin D supplementation on serum LDL levels among people with type 2 diabetes. IV, inverse variance weighted; CI, confidence interval.

**Figure 4:**
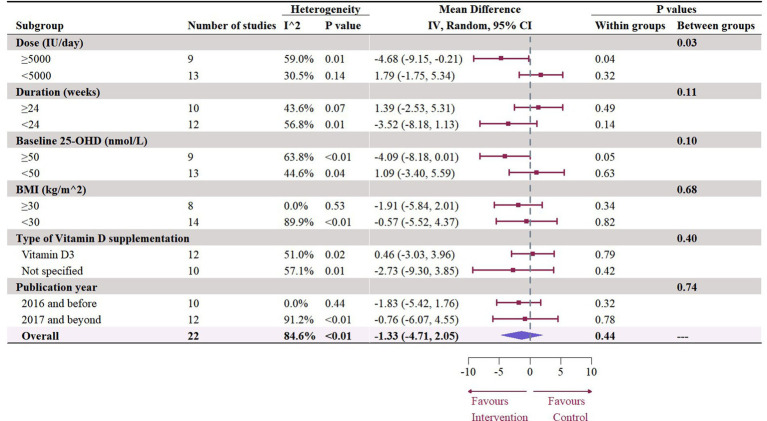
Subgroup analysis of the effect of vitamin D supplementation on serum LDL level in people with type 2 diabetes. IV, inverse variance weighted; CI, confidence interval; 25 OHD, 25-hydroxy vitamin D; BMI, body mass index; IU, international unit.

**Figure 5:**
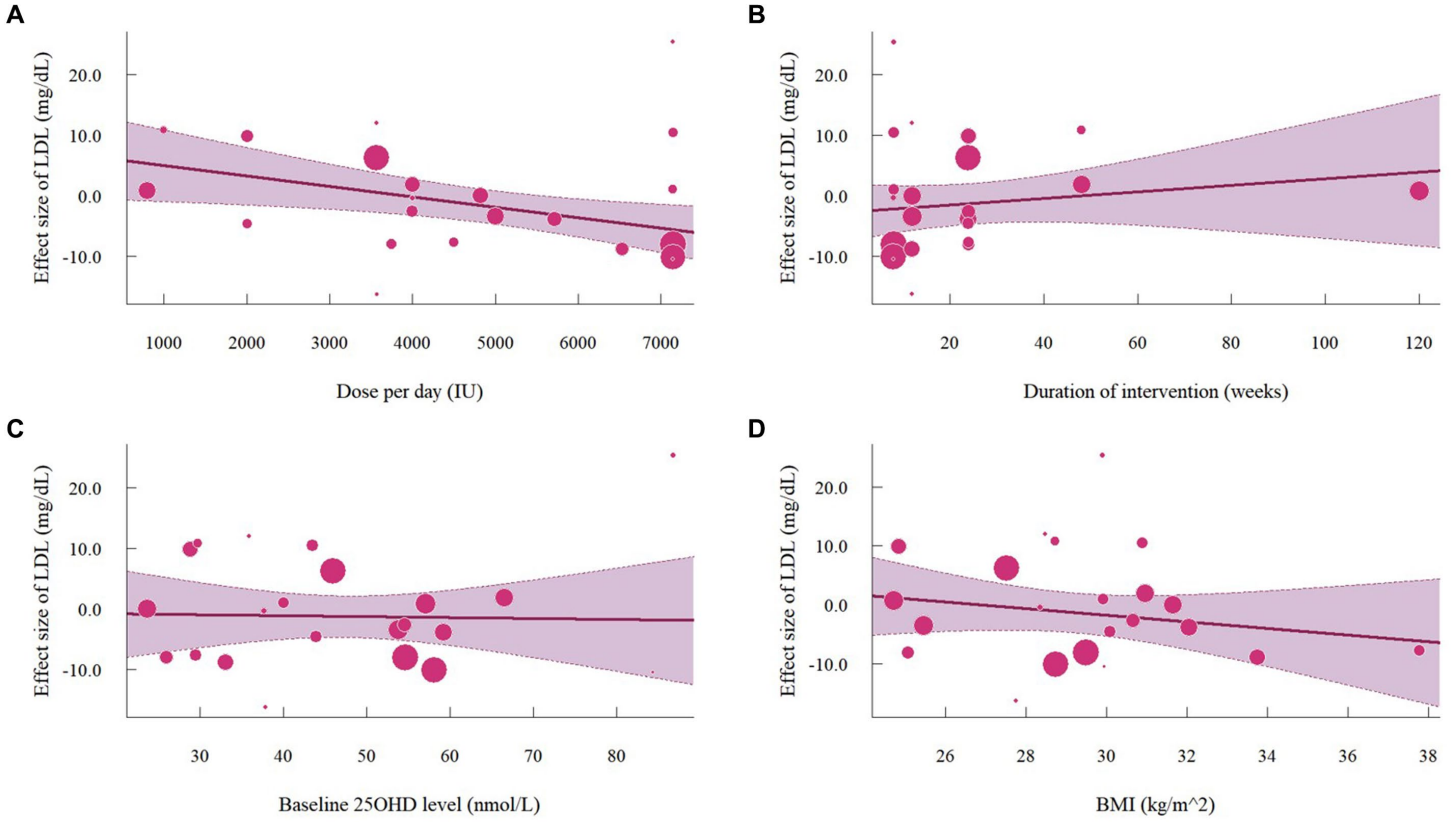
Meta-regressions plots of the effect of vitamin D supplementation on LDL and factors: **(A)** Doses; **(B)** Duration; **(C)** 25 OHD; **(D)** BMI. 25 OHD, 25-hydroxy vitamin D; BMI, body mass index; IU, international unit.

A total of 19 studies with 22 effect sizes (1,633 participants included) reported the data regarding LDL levels. This meta-analysis did not demonstrate any significant effects of vitamin D supplementation on LDL levels (−1.33 mg/dL, 95% CI: −4.71 to 2.05, *p* = 0.44; I^2^ = 84.6%, *p* < 0.01, Figure 3). Nevertheless, the subgroup analyses revealed a significant difference in the effects of vitamin D supplementation among different dosage groups, with a cutoff of 5,000 IU (*p* = 0.03). In studies where daily doses exceeded 5,000 IU, a significant reduction in LDL levels was observed (−4.68 mg/dL, 95% CI: −9.15 to −0.21, *p* = 0.04, Figure 4). Meta-regression analyses revealed a potential association between doses and changes in LDL (estimate: -0.0017, 95% CI: −0.0031 to −0.0004, *p* = 0.01, Figure 5; Supplementary Table S1). A similar finding was observed between dose and effect sizes of LDL when implementing permutation tests (estimate: -0.0017, 95% CI: −0.0032 to −0.0002, *p* = 0.05, Supplementary Table S2).

### 3.4 Effect of vitamin D supplementation on serum HDL level

**Figure 6:**
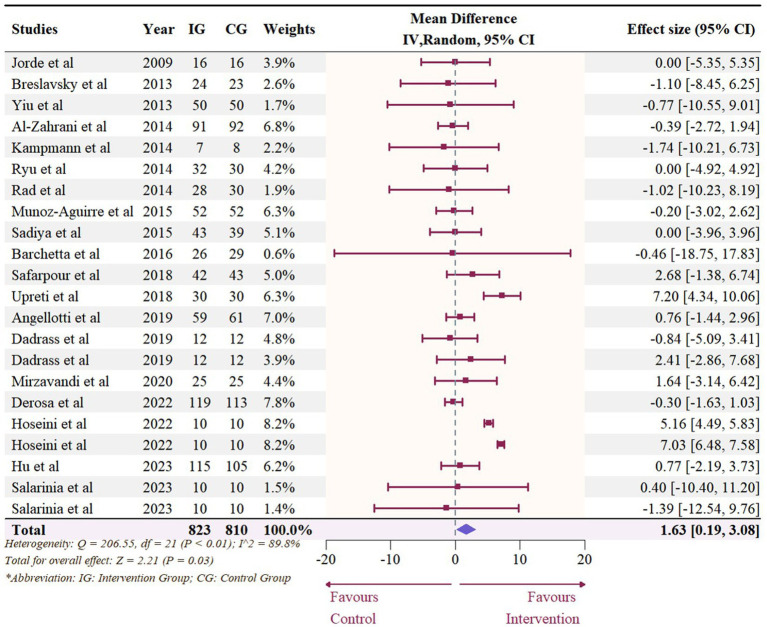
Forest plot of the effect of vitamin D supplementation on serum HDL levels among people with type 2 diabetes. IV, inverse variance weighted; CI, confidence interval.

**Figure 7:**
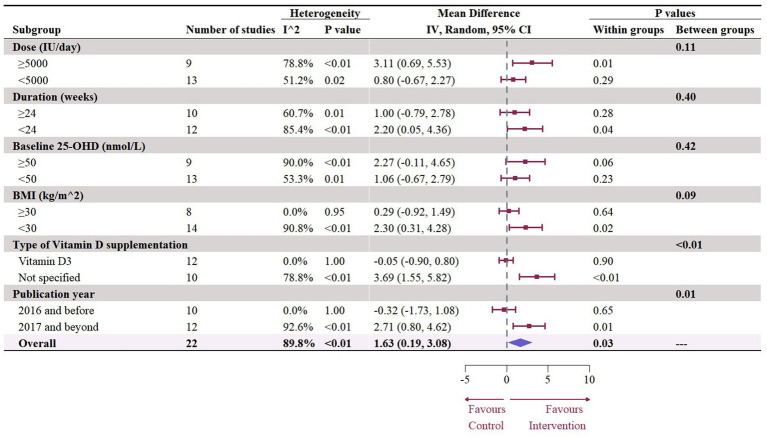
Subgroup analysis of the effect of vitamin D supplementation on serum HDL level in people with type 2 diabetes. IV, inverse variance weighted; CI, confidence interval; 25 OHD, 25-hydroxy vitamin D; BMI, body mass index; IU, international unit.

**Figure 8:**
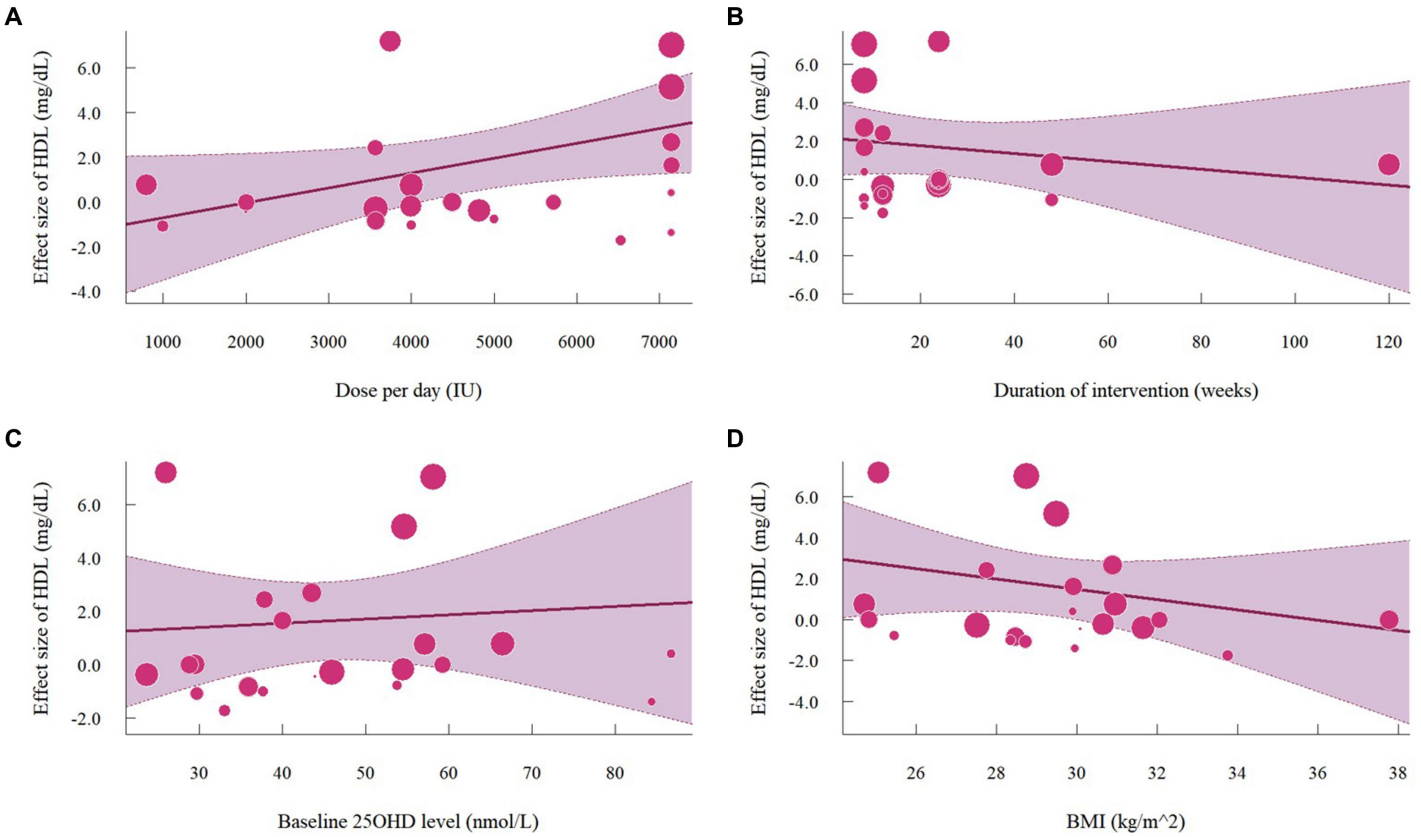
Meta-regressions plots of the effect of vitamin D supplementation on HDL and factors: **(A)** Doses; **(B)** Duration; **(C)** 25 OHD; **(D)** BMI. 25 OHD, 25-hydroxy vitamin D; BMI, body mass index; IU, international unit.

Nineteen studies with 22 effect sizes (1,633 participants) examined the effects of vitamin D supplementation on HDL levels. Overall, vitamin D supplementation significantly increased HDL (1.63 mg/dL, 95% CI: 0.19 to 3.08, *p* = 0.03; I^2^ = 89.8%, *p* < 0.01, Figure 6). For subgroup analyses (Figure 7), studies that did not specify the type of vitamin D supplementation significantly increased HDL (3.92 mg/dL, 95% CI: 1.77 to 6.06, p < 0.01). A significant increase in HDL was also observed in studies published beyond 2017 (2.71 mg/dL, 95% CI: 0.80 to 4.62, *p* = 0.01). Furthermore, doses over 5,000 IU per day significantly increased HDL by 3.11 mg/dL (95% CI: 0.69 to 5.53, p = 0.01) and trials with a duration (< 24 weeks) showed a significant increase in HDL by 2.20 mg/dL (95% CI: 0.05 to 4.36, *p* = 0.04). However, meta-regression analysis (Figure 8; Supplementary Table S1) failed to discover any significant effects on HDL when considering doses, duration, baseline 25OHD and BMI, which was evidenced by the results of permutation tests (Supplementary Table S2).

### 3.5 Effect of vitamin D supplementation on serum TC level

**Figure 9:**
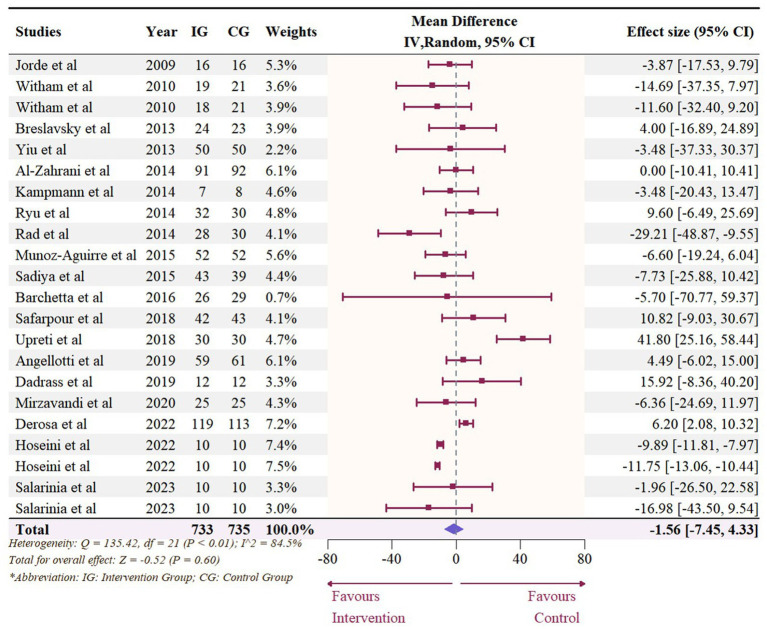
Forest plot of the effect of vitamin D supplementation on serum TC levels among people with type 2 diabetes. IV, inverse variance weighted; CI, confidence interval.

**Figure 10:**
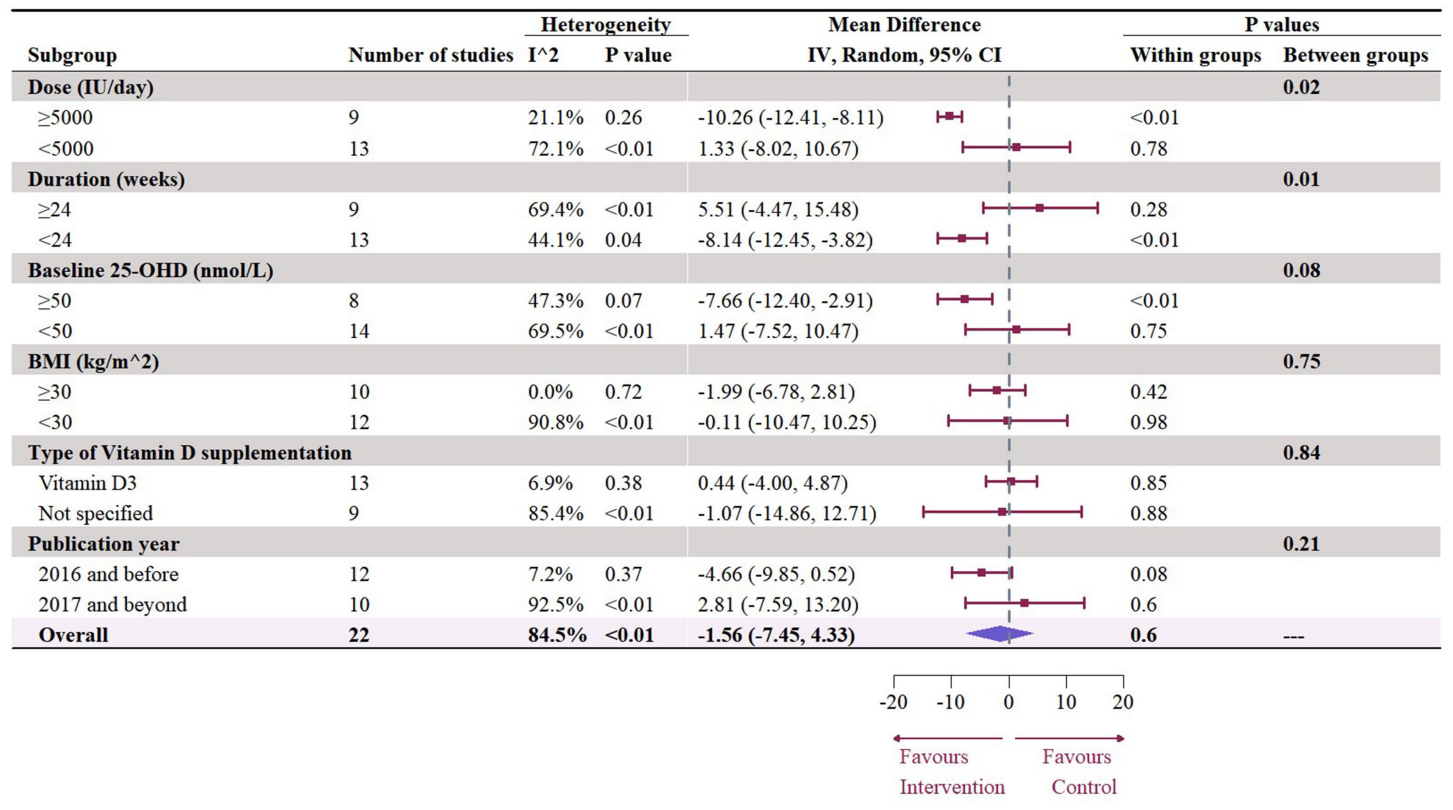
Subgroup analysis of the effect of vitamin D supplementation on serum TC level in people with type 2 diabetes. IV, inverse variance weighted; CI, confidence interval; 25 OHD, 25-hydroxy vitamin D; BMI, body mass index; IU, international unit.

**Figure 11:**
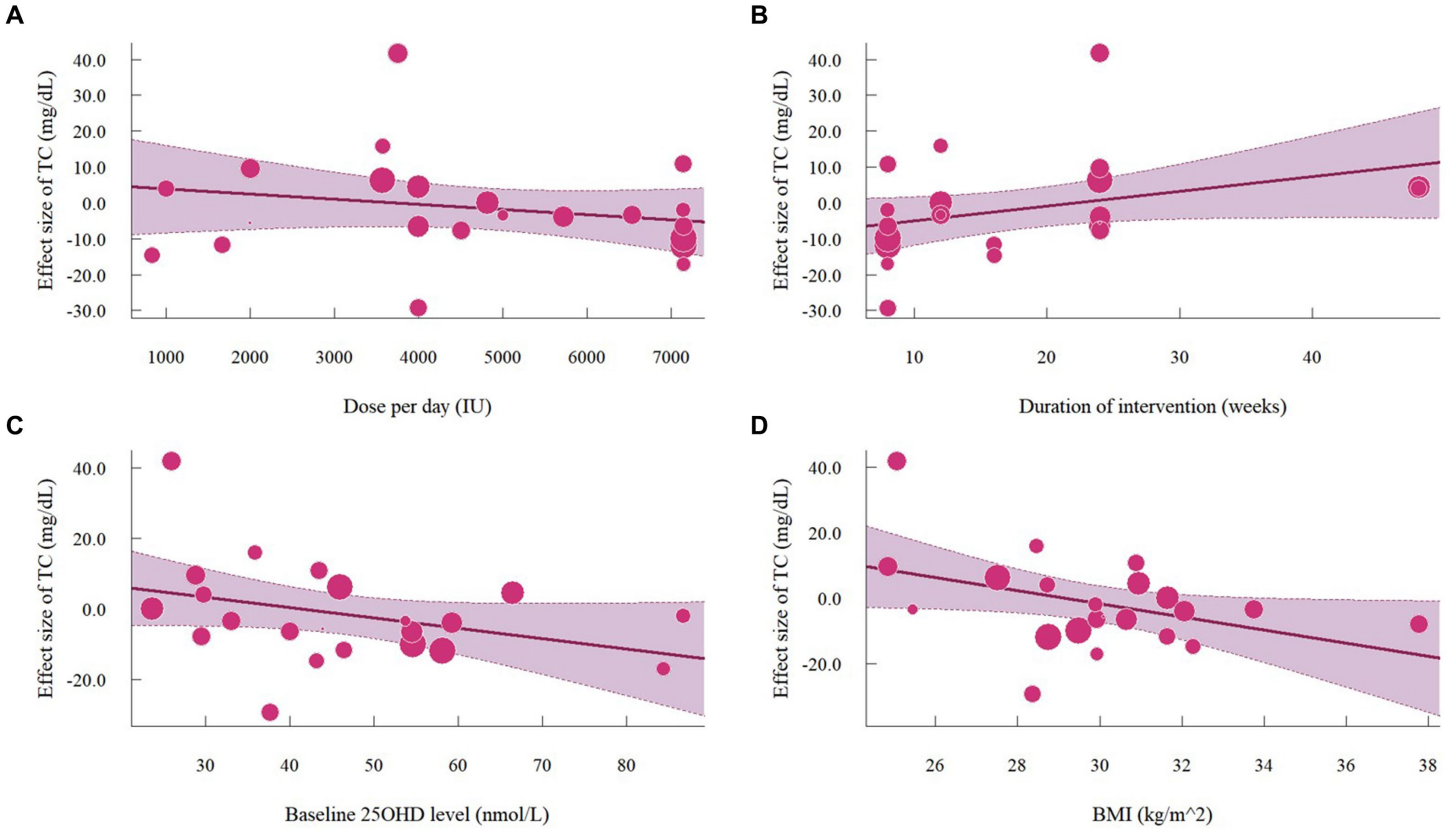
Meta-regressions plots of the effect of vitamin D supplementation on TC and factors: **(A)** Doses; **(B)** Duration; **(C)** 25 OHD; **(D)** BMI. 25 OHD, 25-hydroxy vitamin D; BMI, body mass index; IU, international unit.

A total of 1,468 participants from 19 studies (with 22 effect sizes) provided data in terms of serum TC levels. Results did not demonstrate any significant change in TC (−1.56 mg/dL, 95% CI: −7.45 to 4.33, *p* = 0.60; I^2^ = 84.5%, *p* < 0.01, Figure 9). For subgroup analysis (Figure 10), significant reductions of TC were observed in participants who were administrated vitamin D supplementation for more than 5,000 IU/day (−10.26 mg/dL, 95% CI: −12.41 to −8.11, *p* < 0.01) and received interventions for less than 24 weeks (−8.14 mg/dL, 95% CI: −12.45 to −3.82, *p* < 0.01). Additionally, vitamin D supplementation significantly decreased TC among participants with a baseline 25OHD level over 50 nmol/L (−7.66 mg/dL, 95% CI: −12.40 to −2.91, *p* < 0.01). However, meta-regressions (Figure 11; Supplementary Table S1) did not reveal potential associations in terms of doses (*p* = 0.33) or duration (*p* = 0.08), but indicated a potential linear regression between BMI and TC (estimate: -2.0005, 95% CI: −3.9893 to −0.0116, *p* = 0.05). Permutation tests did not provide any associations between dose, duration, baseline 25 OHD and BMI with TC levels (Supplementary Table S2).

### 3.6 Effect of vitamin D supplementation on serum TG level

**Figure 12:**
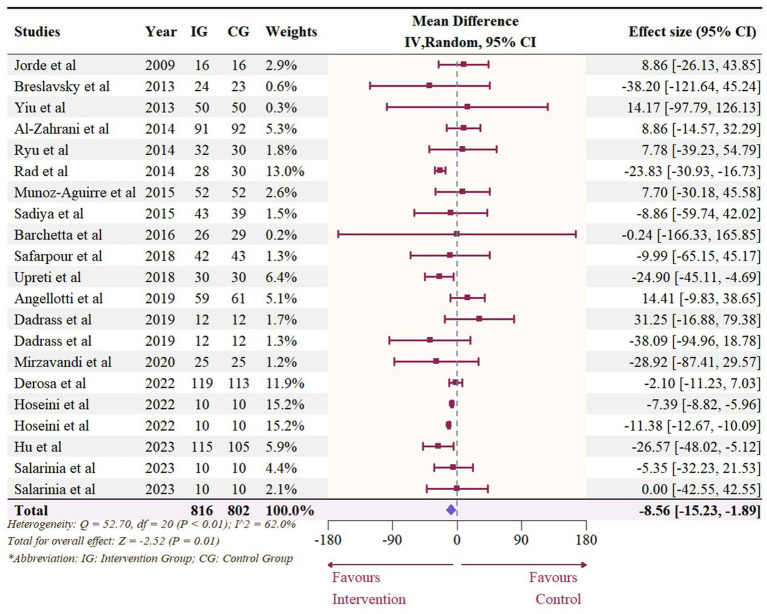
Forest plot of the effect of vitamin D supplementation on serum TG levels among people with type 2 diabetes. IV, inverse variance weighted; CI, confidence interval.

**Figure 13:**
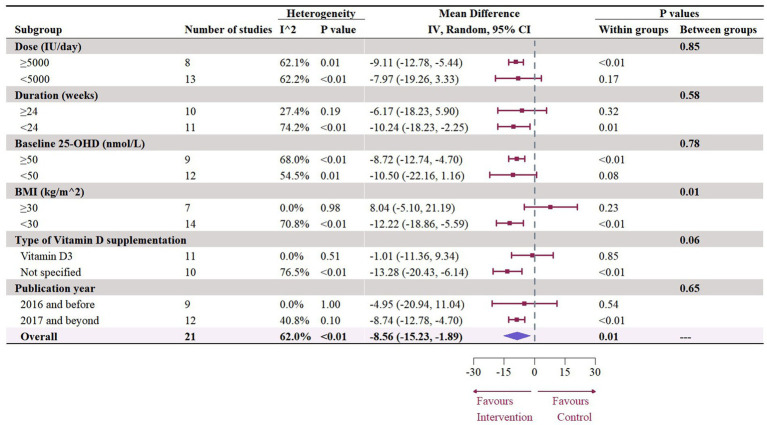
Subgroup analysis of the effect of vitamin D supplementation on serum TG level in people with type 2 diabetes. IV, inverse variance weighted; CI, confidence interval; 25 OHD, 25-hydroxy vitamin D; BMI, body mass index; IU, international unit.

**Figure 14:**
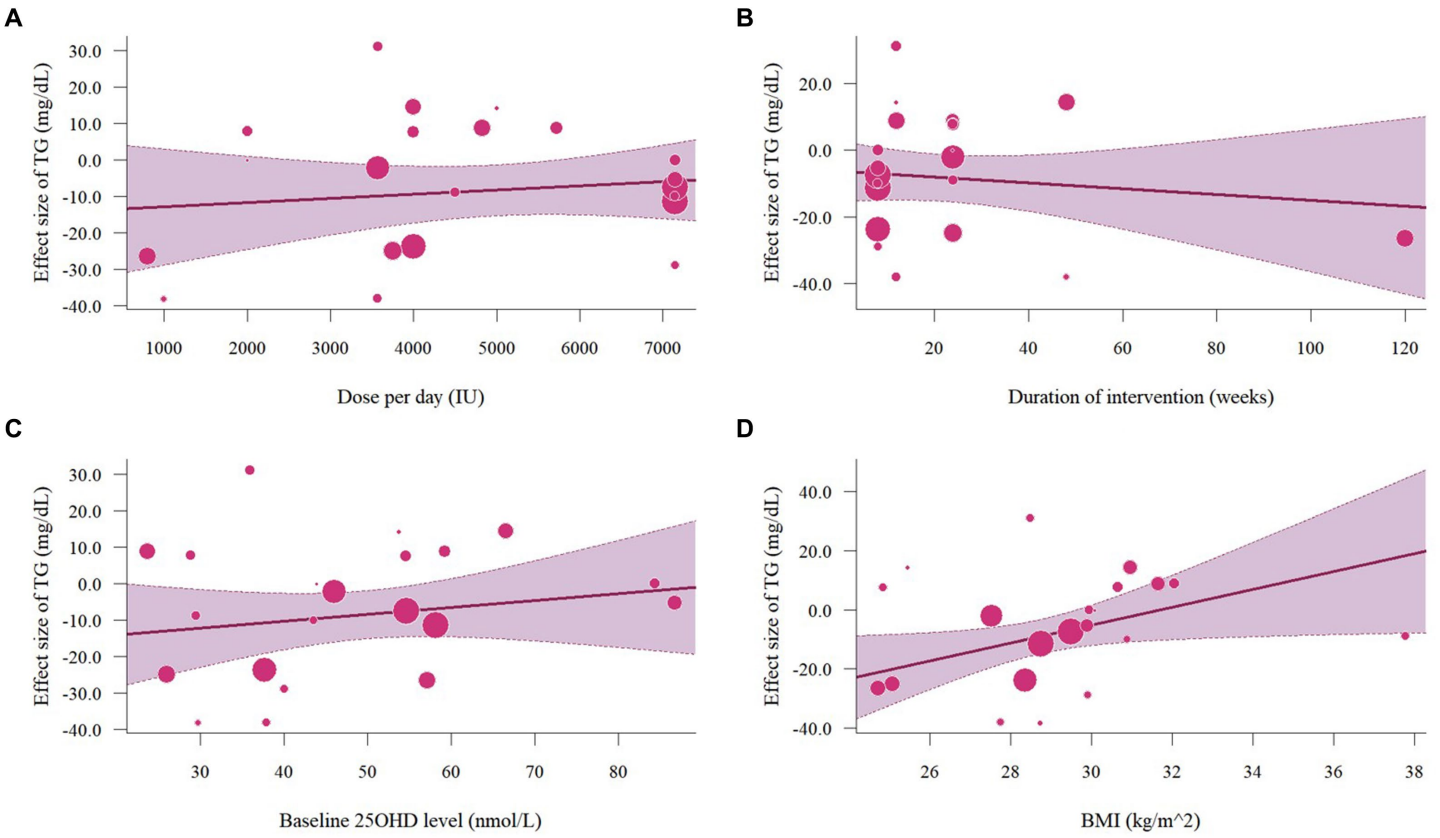
Meta-regressions plots of the effect of vitamin D supplementation on TG and factors: **(A)** Doses; **(B)** Duration; **(C)** 25 OHD; **(D)** BMI. 25 OHD, 25-hydroxy vitamin D; BMI, body mass index; IU, international unit.

The meta-analysis assessed 18 studies (21 effects size) with 1,608 participants that provided data on TG. Results highlighted a significant reduction in TG (−8.56 mg/dL, 95% CI: −15.23 to −1.89, *p* = 0.01; *I*^2^ = 62.0%, *p* < 0.01, Figure 12). For subgroup analysis (Figure 13), a significant decrease of TG was observed in participants with BMI lower than 30 (−12.22 mg/dL, 95% CI: −18.86 to −5.59, *p* < 0.01). Moreover, doses over 5,000 IU/day (−9.11 mg/dL, 95% CI: −12.78 to −5.44, *p* < 0.01) and trial duration less than 24 weeks (−10.24 mg/dL, 95% CI: −18.23 to −2.25, *p* = 0.01) significant reduced TG. Studies that did not specify the type of vitamin D supplementation (−9.71 mg/dL, 95% CI: −13.42 to −5.99, p < 0.01) and published after 2017 demonstrated a significant decrease in TG (−8.74 mg/dL, 95% CI: −12.78 to −4.70, p < 0.01). Meta-regression analysis (Figure 14; Supplementary Table S1) indicated a linear association between BMI and TG (estimate: 3.0285, 95% CI: 0.2065 to 5.8506, *p* = 0.04) but permutation test did not reveal any significant associations (Supplementary Table S2).

### 3.7 Sensitivity analysis

The leave-one-out analyses were conducted and did not find a significant impact on the pooled effect sizes of LDL (Supplementary Figure S1) and TC levels (Supplementary Figure S2) when omitting any single study. The omission of the study conducted by Upreti et al. (38) reduced the overall effect size of TC, but the result was still insignificant. Regarding HDL, the omission of studies conducted by Upreti et al. (38) and two effect sizes from Hoseini et al. (42) resulted in an insignificant overall outcome on HDL (Supplementary Figure S3). In terms of TG (Supplementary Figure S4), leave-one-out analysis revealed that the effects of vitamin D supplementation on TG levels were insignificant after excluding one intervention from Hoseini et al. (42). Given that the changes of effect sizes were not extremely different when omitting one study at a time, the overall results of all meta-analyses were still robust.

### 3.8 Publication bias

Results of publication bias are listed in Supplementary Table S3, and funnel plots are shown in Supplementary Figure S5. No detectable publication bias was found in LDL (Egger’s test: *p* = 0.183; Begg’s test: *p* = 0.756) and TG (Egger’s test: *p* = 0.742; Begg’s test: *p* = 0.507). The funnel plots for LDL and TG showed no apparent asymmetry. However, there was evidence of publication bias in HDL (Egger’s test: *p* = 0.001; Begg’s test: *p* = 0.030) and TC (Egger’s test: *p* = 0.026; Begg’s test: *p* = 0.272), which were also indicated by the presence of asymmetry in their respective funnel plots. Trim-and-fill methods were used to predict the potential missing studies for HDL and TC (Supplementary Table S4). In terms of HDL, when using random effect trim-and-fill methods, 6 studies were statistically added and the overall effect size was still significant (2.11 mg/dL, 95% CI: 0.80 to 3.42, *p* < 0.01). Fixed-effect trim-and-fill method statistically added 11 missing studies and the final effect size was still significant (4.67 mg/dL, 95% CI: 2.72 to 6.63, *p* < 0.01). For TC, although Egger’s test suggested the presence of publication bias in studies on TC, the trim-and-fill method was unable to account for potential missing studies when employing a random effects model. When applying the fix-effect trim-and-fill method, 9 missing studies were statistically added and the final effect size became significant (−10.76 mg/dL, 95% CI: −17.80 to −3.71, *p* < 0.01).

## 4 Discussions

In this meta-analysis, we included 20 RCTs investigating the effects of vitamin D supplementation on lipid profiles in people with type 2 diabetes. The findings showed a significant increase in HDL and a decrease in TG levels. However, no significant impact was observed on LDL and TC levels. Our results are similar to the findings from previous meta-analyses. One meta-analysis on the general population demonstrated significant improvement of vitamin D in TC, LDL, and TG levels, while not in HDL levels (45). For the population with pre-diabetes, a meta-analysis revealed a significant decrease in TG levels, but no significant change in LDL, HDL, and TC levels (46). Nevertheless, a meta-analysis conducted on adults with metabolic syndrome failed to find any significant alteration in LDL, HDL, TC, and TG after the implementation of vitamin D supplementation (47). Therefore, the effects of vitamin D on lipid profiles may vary depending on the specific conditions that participants are afflicted with.

The previous meta-analysis of the same topic by Jafari et al. (6) in 2016 reported improvements in TG and TC levels but not LDL and HDL levels. The differences may be explained by the various application of vitamin D. It is worth noting, that the previous meta-analysis included trials employing both fortification and supplementation. In their meta-analysis, all studies that applied vitamin D fortification were less than 2000 IU/day but demonstrated enhanced effects on lipid profiles (6). The absorption of vitamin D is optimized when consumed in conjunction with foods rich in dietary fat (48), and vitamin D fortification is often taken with fat-containing foods (49, 50), which may explain why vitamin D fortification may yield different results compared to vitamin D supplementation. Additionally, we examined the differences among studies published before and after 2017. For studies before 2017, our findings align with the subgroup analysis results reported by Jafari et al. (6). However, studies after 2017 indicated a potential to improve HDL and TG but not LDL and TC. Intriguingly, our findings from subgroup analyses by type also reported similar outcomes. Among the studies that did not specify the type of vitamin D supplementation, five studies were published after 2017. In this sense, the observed enhancement in studies after 2017 could potentially be linked to the unspecified type. It may, however, raise concerns about the potential overestimation of true effects due to the lack of reporting on the specific type.

Subgroup analyses under doses revealed a significant improvement across lipid profiles with the administration of higher doses. Both meta-regressions and permutation tests confirmed the positive effect of doses in improving LDL levels, thereby reinforcing the robustness of its association. Considering that the current aim of treating and preventing dyslipidemia is to improve serum LDL levels (51), our meta-analysis highlights the potential of higher doses of vitamin D supplementation in reducing dyslipidemia. Additionally, potential improvements in lipid profiles are more likely to occur in shorter trial duration (< 24 weeks). Similar results were demonstrated in many previous meta-analyses, which may be attributed to reduced adherence to vitamin D administration in long-term trials (6, 45, 52, 53). Moreover, considering the half-life of vitamin D is approximately 2 months, its therapeutic effects are typically achieved and maintained within a short period (53). Nevertheless, only 3 studies implemented interventions for 48 weeks (12, 29, 40). Consequently, there is still a dearth of evidence regarding the impact of longer durations.

Moreover, participants with a baseline BMI < 30 kg/m^2^ are more likely to derive lipid profile improvements from vitamin D supplementation, especially on TG levels. Due to the fat-soluble nature of vitamin D, it is more likely to be sequestrated in adipose tissue (54). Thus, individuals with a lowerBMI may exhibit a higher concentration of 25 OHD serum level when administered the same dosage of vitamin D supplementation, compared to people with a higher BMI. However, permutation tests indicated that the potential associations between BMI and TC or TG may be susceptible to false positive findings. The permutation test is aimed to control the type 1 error rate, and thus often results in higher *p* values compared to meta-regressions (24). Also, participants with 25 OHD levels of more than 50 nmol/L experienced significant improvements in TC and TG levels. Jafari et al. (6) previously discovered that vitamin D may be more effective in improving TC and LDL among people with T2D and insufficient or sufficient serum vitamin D status. This may be because only four studies regarding 25 OHD ≥ 50 nmol/L were included in their meta-analysis (6).

In this current study, inconsistent results in subgroup analysis and meta-regression analysis were only observed regarding TC, which may be due to the impact of outliers (55). Sensitivity analyses revealed that the study by Upreti et al. (38) and two effect sizes from Hoseini et al. (42) were more likely to be considered as potential outliers. In the study of Upreti et al. (38), 60 participants (30 in each arm) were recruited in a 24-week intervention. The TC levels between the intervention group and the control group at baseline were considerably different (no statistical analysis was performed), but were similar after the intervention. As such, this significant increase in TC was more likely attributed to the difference in baseline TC levels rather than the effects of vitamin D. Hoseini et al. (42) conducted an 8-week single-blinded RCT using aerobic training (AT) and vitamin D supplementation comprised four groups (AT+ vitamin D; AT; Vitamin D; placebo). The baseline lipid profiles were comparable among the four groups; however, the significant effects of vitamin D supplementation on lipid profiles may vary due to the limited number of participants included (10 in each arm).

Publication biases were observed in HDL and TC, thereby the trim-and-fill method was performed. All hypothetical compensated studies demonstrated favorable impacts on HDL and TC levels. Most studies analyzed for TC overlapped with those in the analysis of TG and LDL, and the studies examining the effects on HDL were identical to those used for LDL assessment. Given that no publication bias was detected in terms of LDL and TG, it seemed unlikely that the absences of compensated studies in HDL and TC were caused by publication bias. Rather, the asymmetry discovered in funnel plots was more likely caused by other factors such as high heterogeneity between studies or different quality of studies (56). Notably, since the heterogeneity of meta-analyses regarding HDL and TC was relatively high, the results of the trim-and-fill method may not be accurate (56).

There are some plausible mechanisms through which vitamin D could potentially modulate HDL and TG. Firstly, vitamin D could potentially inhibit the expression of nuclear factor sterol regulatory element-binding protein 1c, which plays a role in hepatic triglyceride synthesis (46). Additionally, vitamin D may upregulate lipoprotein lipase (LPL) in muscle and fat tissues. A cross-sectional study has shown a positive association between serum vitamin D levels with LPL (57), and activation of LPL would further increase clearance of circulating lipoprotein particles (6, 46). Third, increasing vitamin D serum levels may improve TG and HDL in people with T2D. Vitamin D deficiency potentially impacts the functioning of beta cells and insulin resistance, consequently affecting lipoprotein metabolism and leading to elevated TG levels and decreased HDL levels (58). Lastly, vitamin D may reduce TG levels by regulating parathyroid hormone (PTH). The elevation of PTH levels may lead to an increase in the production of TG, and the presence of vitamin D inhibits the secretion of PTH in the bloodstream (58, 59).

A strength of this meta-analysis is that most included studies sustained a moderate to high quality, with low drop-out rates and followed per-protocol analyses. Thus, our study highlighted the value of vitamin D supplementation in the management of CVD, particularly in its role in regulating dyslipidemia, among people with T2D. Moreover, permutation tests were applied to enhance the robustness of our results. However, there are some limitations which should be further considered. Firstly, the heterogeneity between studies was high. It may be due to dissimilar baseline lipid profiles among participants, different study designs of interventions, and various ethnicities of included populations. Second, due to the lack of baseline BMI or 25 OHD data, not all potentially eligible studies were included in this meta-analysis, which may affect the generalizability of the outcome.

## 5 Conclusion

In conclusion, this meta-analysis demonstrated that vitamin D supplementation can significantly increase HDL levels and decrease TG levels among people with type 2 diabetes. However, vitamin D supplementation failed to improve LDL and TC levels. The potential benefits of lipid profiles from vitamin D supplementation are more likely to be observed with shorter intervention periods or higher doses of vitamin D administration. While some potential effects of vitamin D supplementation have been noted in this study, additional long-term and rigorous RCTs are required to validate clinical significance. Moreover, further studies should also consider the influence of factors such as doses, type of vitamin D supplementation, participants’ BMI and serum 25OHD levels on the effects of vitamin D on lipid profiles among people with T2D.

## Data availability statement

The original contributions presented in this study are included in the article/Supplementary material, further inquiries can be directed to the corresponding author.

## Author contributions

QLu: Conceptualization, Data curation, Formal analysis, Investigation, Methodology, Software, Supervision, Validation, Visualization, Writing – original draft, Writing – review & editing. QLi: Methodology, Validation, Writing – original draft, Writing – review & editing. YX: Data curation, Methodology, Writing – original draft.
