# Supplementary material for: The effects of vitamin D supplementation on serum lipid profiles in people with type 2 diabetes: a systematic review and meta-analysis of randomized controlled trials
Source: Front Nutr. 2024 Jun 5;11:1419747. doi: 10.3389/fnut.2024.1419747 (PMC11188582; doi:10.3389/fnut.2024.1419747)
Supplement: Supplementary file 1 [file Data_Sheet_1.docx]

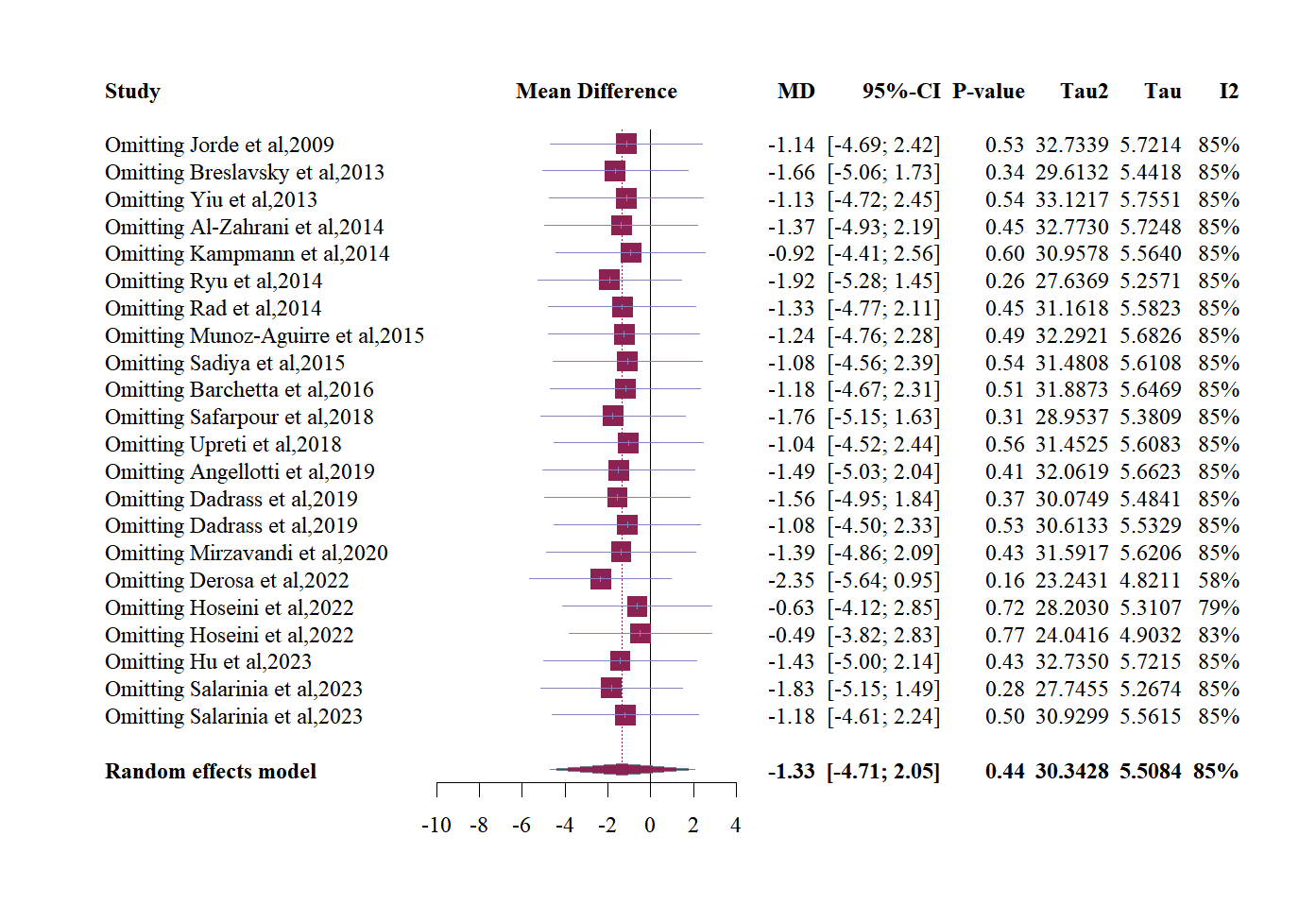
Supplementary Material

**Supplementary Figure 1.** The forest plot of leave-one-out analysis of vitamin D supplementation of serum **LDL** level. *Abbreviations:* MD: mean difference; CI: confidence interval.

**Supplementary Figure 2.**
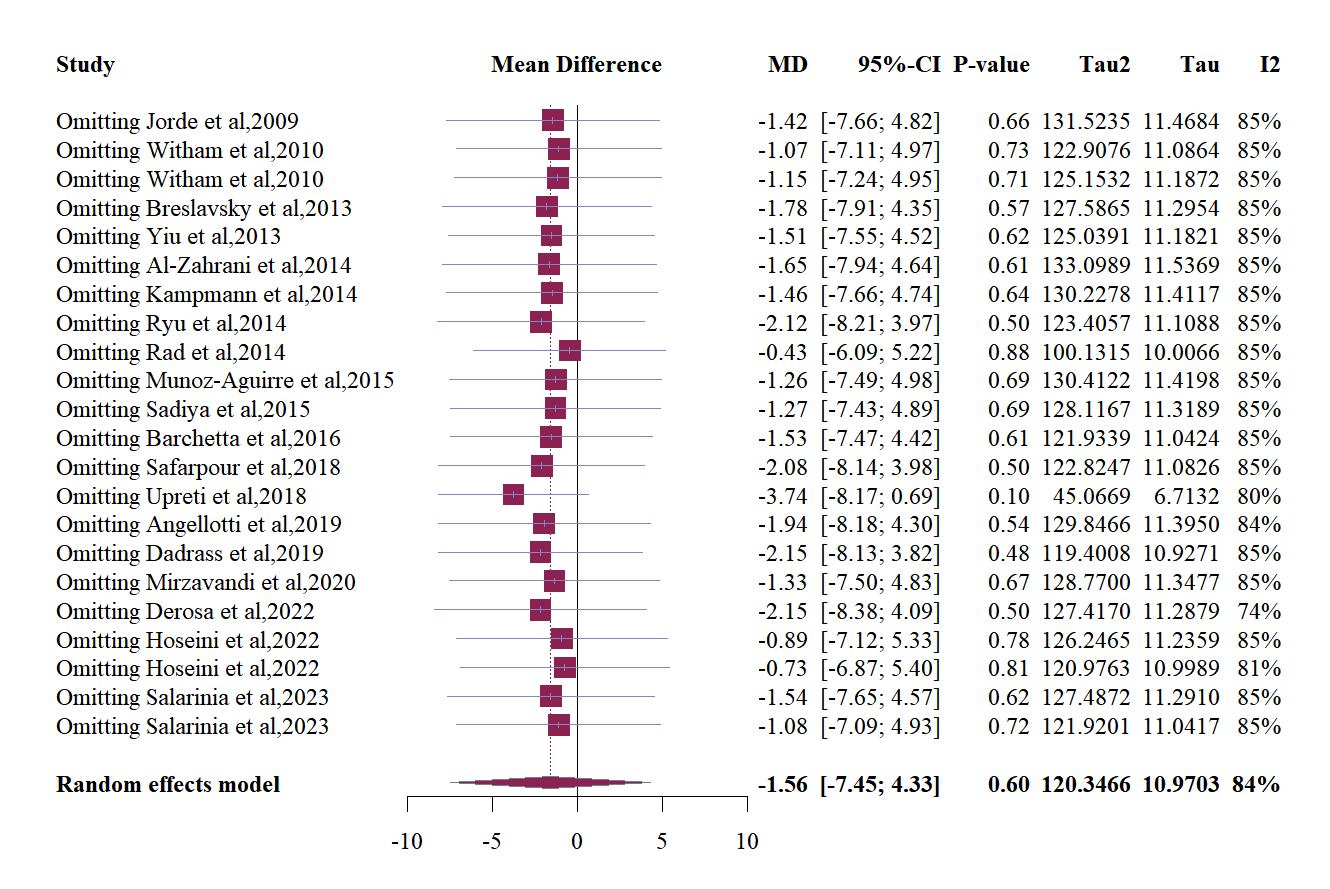
 The forest plot of leave-one-out analysis of vitamin D supplementation of serum **TC** level. *Abbreviations:* MD: mean difference; CI: confidence interval.


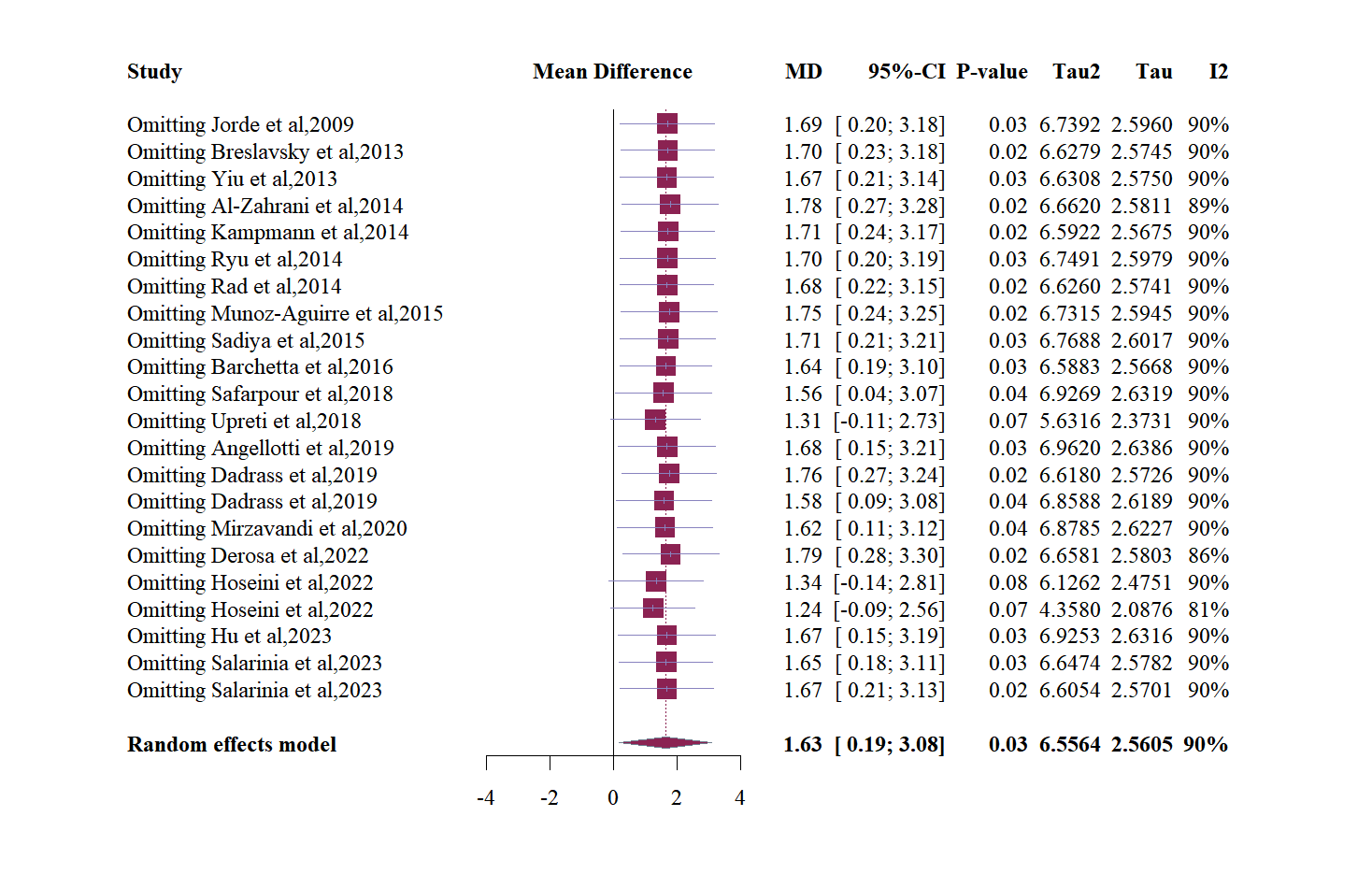
 **Supplementary Figure 3.** The forest plot of leave-one-out analysis of vitamin D supplementation of serum **HDL** level. *Abbreviations:* MD: mean difference; CI: confidence interval.

**Supplementary Figure**
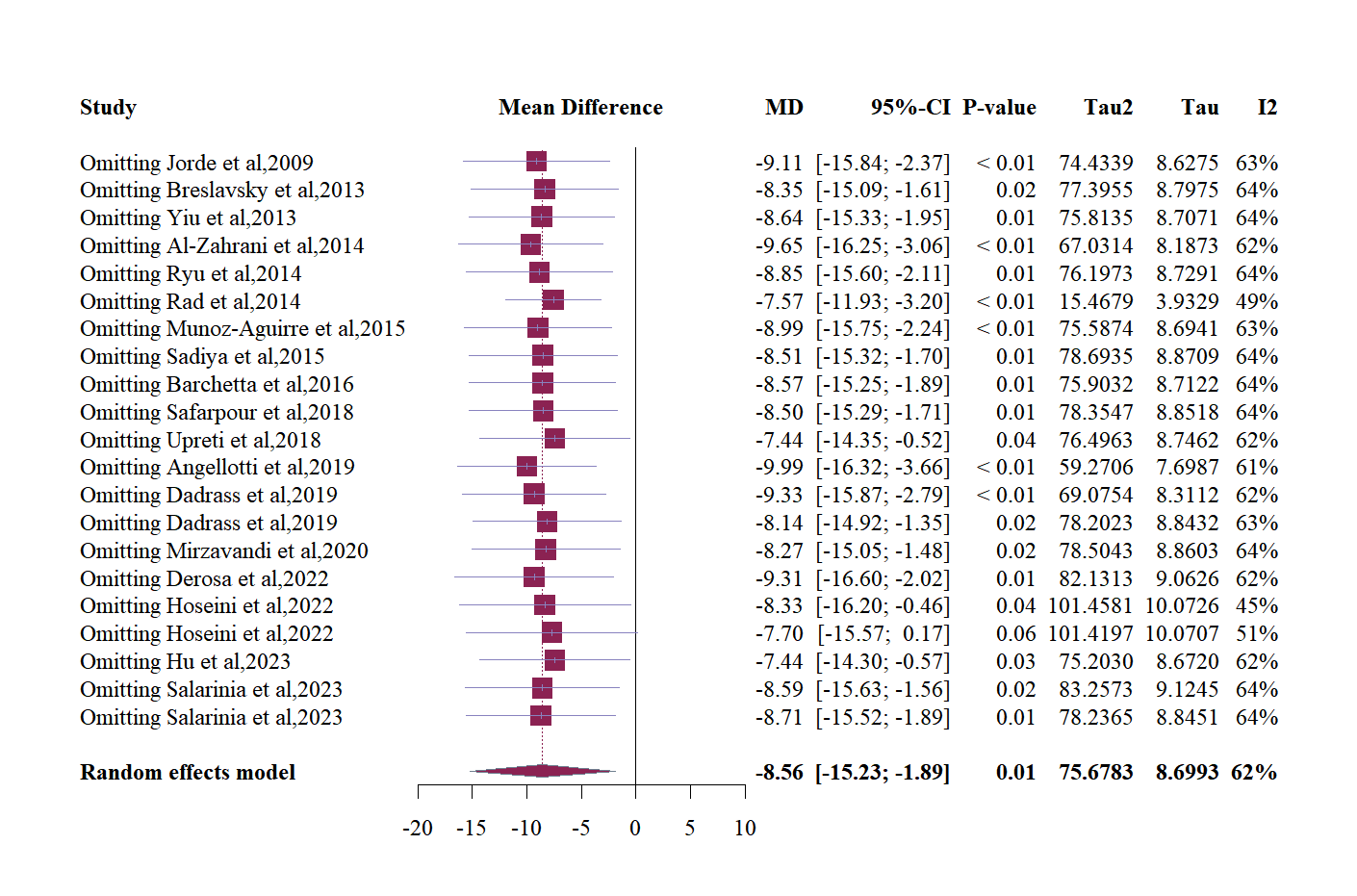
**4.** The forest plot of leave-one-out analysis of vitamin D supplementation of serum **TG** level. *Abbreviations:* MD: mean difference; CI: confidence interval.


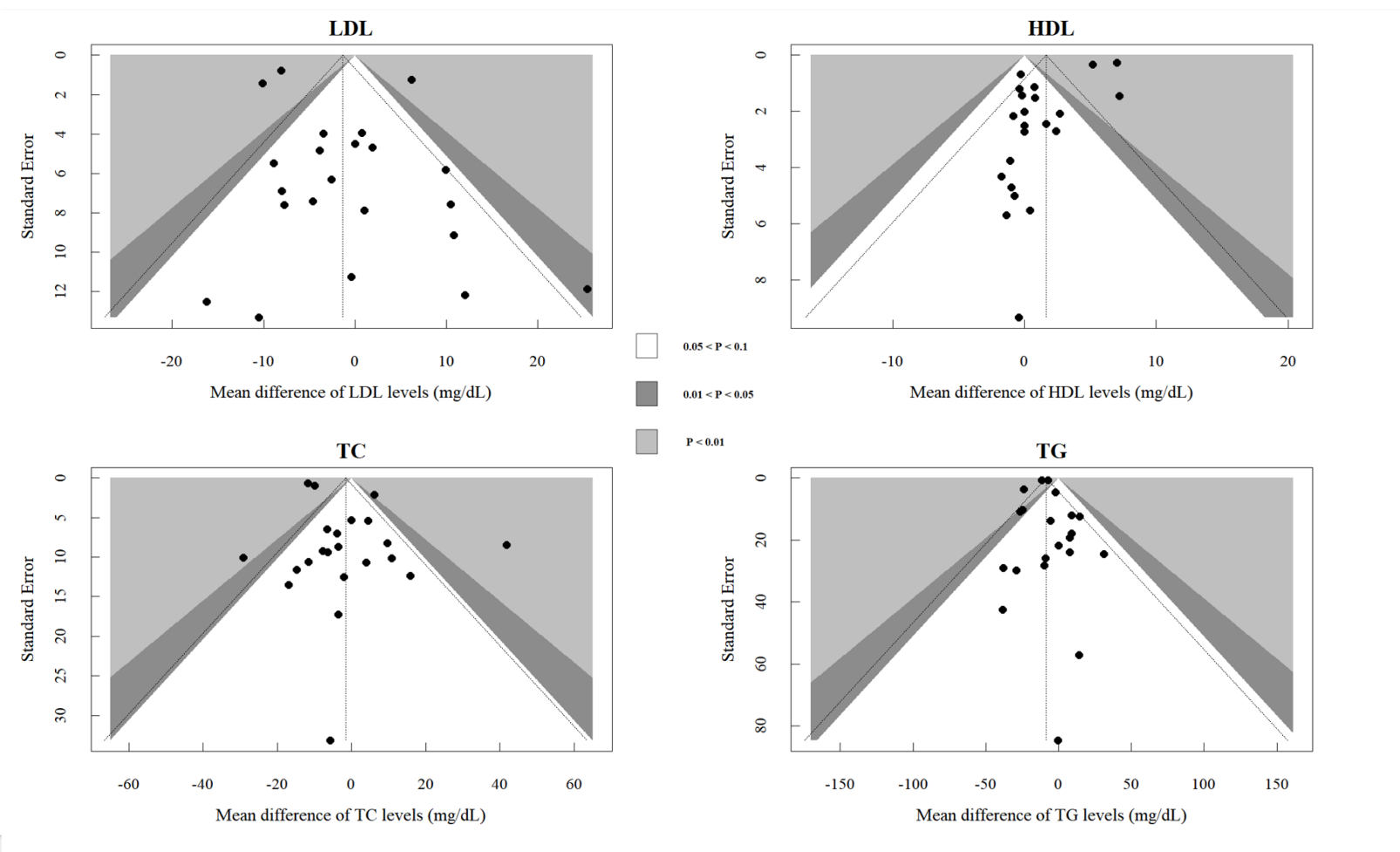
**Supplementary Figure** **5.** Funnel plots of the effect of vitamin D supplementation on serum **lipid profiles** in people with type 2 diabetes. *Abbreviations:* LDL: Low-density lipoprotein; HDL: high-density lipoprotein; TC: total cholesterol; TG: triglyceride.

**Supplementary Table 1.** The results of meta-regression analyses in terms of doses, duration, baseline 25 OHD and BMI.

| **Subgroups** | **Estimate** | **Lower limit** | **Upper limit** | **P value** |
| --- | --- | --- | --- | --- |
| **LDL** |  |  |  |  |
| Dose (IU/day) | -0.0017 | -0.0031 | -0.0004 | 0.013 |
| Duration (weeks) | 0.0542 | -0.0682 | 0.1766 | 0.386 |
| Baseline 25OHD level (nmol/L) | -0.0155 | -0.2539 | 0.2229 | 0.899 |
| BMI (kg/m^2^) | -0.5607 | -1.6944 | 0.5729 | 0.332 |
|  |  |  |  |  |
| **HDL** |  |  |  |  |
| Dose (IU/day) | 0.0007 | -0.0000 | 0.0013 | 0.051 |
| Duration (weeks) | -0.0205 | -0.0741 | 0.0330 | 0.452 |
| Baseline 25OHD level (nmol/L) | 0.0158 | -0.0829 | 0.1145 | 0.754 |
| BMI (kg/m^2^) | -0.2516 | -0.7260 | 0.2227 | 0.298 |
|  |  |  |  |  |
| **TC** |  |  |  |  |
| Dose (IU/day) | -0.0014 | -0.0043 | 0.0014 | 0.326 |
| Duration (weeks) | 0.4112 | -0.0509 | 0.8734 | 0.080 |
| Baseline 25OHD level (nmol/L) | -0.2926 | -0.6452 | 0.0600 | 0.100 |
| BMI (kg/m^2^) | -2.0005 | -3.9893 | -0.0116 | 0.049 |
|  |  |  |  |  |
| **TG** |  |  |  |  |
| Dose (IU/day) | 0.0011 | -0.0024 | 0.0047 | 0.531 |
| Duration (weeks) | -0.0873 | -0.3459 | 0.1713 | 0.508 |
| Baseline 25OHD level (nmol/L) | 0.1892 | -0.2412 | 0.6196 | 0.389 |
| BMI (kg/m^2^) | 3.0285 | 0.2065 | 5.8506 | 0.035 |

*Abbreviations:* LDL: Low-density lipoprotein; HDL: high-density lipoprotein; TC: total cholesterol; TG: triglycerides; 25 OHD: 25-hydroxy vitamin D; BMI: body mass index.

**Supplementary Table 2.** The results of permutation tests in terms of doses, duration, baseline 25 OHD and BMI.

| **Subgroups** | **Estimate** | **Lower limit** | **Upper limit** | **P value** |
| --- | --- | --- | --- | --- |
| **LDL** |  |  |  |  |
| Dose (IU/day) | -0.0017 | -0.0032 | -0.0002 | 0.05 |
| Duration (weeks) | 0.0542 | -0.0743 | 0.1827 | 0.45 |
| Baseline 25OHD level (nmol/L) | -0.0155 | -0.2661 | 0.2351 | 0.90 |
| BMI (kg/m^2^) | -0.5607 | -1.7430 | 0.6215 | 0.36 |
|  |  |  |  |  |
| **HDL** |  |  |  |  |
| Dose (IU/day) | 0.0007 | 0.0001 | 0.0013 | 0.11 |
| Duration (weeks) | -0.0205 | -0.0679 | 0.0268 | 0.43 |
| Baseline 25OHD level (nmol/L) | 0.0158 | -0.0716 | 0.1031 | 0.78 |
| BMI (kg/m^2^) | -0.2516 | -0.6665 | 0.1632 | 0.32 |
|  |  |  |  |  |
| **TC** |  |  |  |  |
| Dose (IU/day) | -0.0014 | -0.0045 | 0.0016 | 0.37 |
| Duration (weeks) | 0.4112 | -0.0823 | 0.9047 | 0.10 |
| Baseline 25OHD level (nmol/L) | -0.2926 | -0.6645 | 0.0793 | 0.13 |
| BMI (kg/m^2^) | -2.0005 | -4.0726 | 0.0717 | 0.07 |
|  |  |  |  |  |
| **TG** |  |  |  |  |
| Dose (IU/day) | 0.0011 | -0.0023 | 0.0046 | 0.54 |
| Duration (weeks) | -0.0873 | -0.3313 | 0.1567 | 0.53 |
| Baseline 25OHD level (nmol/L) | 0.1892 | -0.2299 | 0.6084 | 0.43 |
| BMI (kg/m^2^) | 3.0285 | 0.3933 | 5.6638 | 0.10 |

*Abbreviations:* LDL: Low-density lipoprotein; HDL: high-density lipoprotein; TC: total cholesterol; TG: triglycerides; 25 OHD: 25-hydroxy vitamin D; BMI: body mass index.

**Supplementary Table 3.** The results of publication bias detection by using Begg’s test and Egger’s test.

| Publication Bias (p-value) | | |
| --- | --- | --- |
|  | Begg’s Test | Egger’s Test |
| LDL | 0.756 | 0.183 |
| HDL | 0.030 | 0.001 |
| TC | 0.272 | 0.026 |
| TG | 0.507 | 0.742 |

*Abbreviations:* LDL: Low-density lipoprotein; HDL: high-density lipoprotein; TC: total cholesterol; TG: triglycerides.

|  | **No. of missing studies** | **Total No. of studies** | **I^2^** | **P of heterogeneity** | **Effect size** | **95% CI** | **P value** |
| --- | --- | --- | --- | --- | --- | --- | --- |
| **HDL** |  |  |  |  |  |  |  |
| Original | 0 | 22 | 89.8% | <0.01 | 1.63 | (0.19 to 3.08) | 0.03 |
| Trim-and-fill (Random effect) | 6 | 28 | 86.9% | <0.01 | 2.11 | (0.80 to 3.42) | <0.01 |
| Trim-and-fill (Fixed effect) | 11 | 33 | 91.7% | <0.01 | 4.67 | (2.72 to 6.63) | <0.01 |
|  |  |  |  |  |  |  |  |
| **TC** |  |  |  |  |  |  |  |
| Original | 0 | 22 | 84.5% | <0.01 | -1.56 | (-7.45 to 4.33) | 0.60 |
| Trim-and-fill (Random effect) | 0 | 22 | 84.5% | <0.01 | -1.56 | (-7.45 to 4.33) | 0.60 |
| Trim-and-fill (Fixed effect) | 9 | 31 | 89.4% | <0.01 | -10.76 | (-17.80 to -3.71) | <0.01 |

**Supplementary Table 4.** The results of trim-and-fill methods by using fixed-effect model and random-effects model for overall effects of high-density lipoprotein (HDL) and total cholesterol (TC).

*Abbreviations:* *No*.: Number; *CI:* confidence interval.
